# Application of Product of Vitrification of Asbestos-Cement Waste and CRT Glass Cullet as Reinforcing Phase in Surface Composites Produced by FSP Method

**DOI:** 10.3390/ma17225508

**Published:** 2024-11-12

**Authors:** Józef Iwaszko, Krzysztof Kudła, Małgorzata Lubas

**Affiliations:** 1 Faculty of Production Engineering and Materials Technology, Department of Materials Engineering, Czestochowa University of Technology (CUT), 19 Armii Krajowej Ave., 42-200 Czestochowa, Poland; malgorzata.lubas@pcz.pl; 2 Faculty of Mechanical Engineering and Computer Science, Czestochowa University of Technology (CUT), 21 Armii Krajowej Ave., 42-200 Czestochowa, Poland; krzysztof.kudla@pcz.pl

**Keywords:** asbestos-cement waste, CRT glass cullet, aluminum alloy, metal matrix composite, vitrification, friction stir processing

## Abstract

In this study, the vitrification of asbestos-cement waste (ACW) and glass cullet from cathode-ray tubes (CRTs) was performed. The resulting product of vitrification from the abovementioned waste was used as the reinforcing phase in a composite with the AA7075 alloy matrix. The composite was made by means of the FSP (friction stir processing) method. The main aim of this work was to determine whether the product of the vitrification can be utilized as the reinforcing phase in the composite. The tests show that introducing the vitrification product into the composite matrix increases both the hardness of the material and its wear resistance. The composite was characterized by a 39% higher hardness and 30.4% higher wear resistance compared to the initial AA7075 alloy. The changes in the properties were caused by strong refinement of the grains, but primarily by the presence of the hard particles of the reinforcing phase in the composite matrix. This research demonstrates that vitrified material, thanks to its properties, can constitute a full-value reinforcing material that can ultimately replace more expensive engineering materials in composites.

## 1. Introduction

The selection of materials is one of the key aspects of any production process. In recent years, increasingly more attention has been paid to ensure that this choice fits into the sustainable development strategy. The desire to reduce the consumption of natural resources forces us to look for technological and material solutions that will burden the environment as little as possible. Therefore, waste or recycled materials are increasingly used in production processes [1,2]. The possibility of limiting the exploitation of natural resources through effective waste management and restoring their utility functions is a manifestation of concern for the natural environment. Such actions are particularly justified in relation to materials harmful to the environment. One such material is asbestos-cement waste (ACW) [3]. Although most countries have stopped producing asbestos products [4,5], the problem with asbestos is still current as this material occurs in many products still in use. As published data indicate, this waste can be neutralized, for example, by thermal, chemical or biological methods [6,7,8]. Vitrification is particularly effective in the disposal of asbestos-cement waste [9,10,11]. This is a heat treatment process during which organic components decompose, while inorganic components from the material undergoing vitrification migrate into the glass structure [12]. As a result of vitrification, an amorphous material with properties typical of glass is obtained. The application potential of the vitrification product is shaped primarily by its high chemical resistance and hardness, which allows its use, e.g., in the building materials industry or in road construction, as a substitute for natural raw materials. Unfortunately, vitrification is a high-temperature process, which is associated with the high costs of this treatment [13]. This problem can be partially solved by rationalizing processing, combining, for example, asbestos disposal with the disposal of other waste and using the influence of the mutual interaction of the components. Preliminary research conducted by the authors of this work showed that, for example, the simultaneous vitrification of asbestos-cement waste and glass cullet from used CRTs is possible [11].

An important factor that should influence the choice of the waste processing method is the application potential of the resulting product. Vitrified material is most often used in the production of various types of building materials, e.g., concrete additives, or in road construction as a substitute for natural aggregates [14,15]. Taking into account the properties of vitrified materials, especially their chemical resistance and high hardness, the potential of these materials is not fully exploited. According to the authors of this work, vitrified materials are suitable for use in the production of modern engineering materials with a wide range of applications, including metal matrix composites. This was the main aim of this work, namely, to determine whether asbestos-cement waste and CRT cullet subjected to vitrification can be used in the production of composites as a phase reinforcing the matrix of these composites.

The novelty of this work is the first use of the vitrification product of asbestos-cement waste and CRT cullet as the reinforcing phase in a surface composite with a metal matrix, combined with an assessment of the effectiveness of such a solution. To the best of the knowledge of the authors of this work, there are no analogous studies in the literature or reports on the use or even the possibility of using the vitrification product of the abovementioned products in the production of this type of composite materials. This fact prompted the authors of this work to conduct their own research.

## 2. Materials and Research Methodology

The matrix of the designed composite was AA7075 (Al-5.5Zn-2.4Mg-1.6Cu-0.20Cr) aluminum alloy in the T6 state, and the reinforcing phase was a product of the vitrification of asbestos-cement waste and glass from a CRT.

In the vitrification process, a fragment of corrugated asbestos-cement board used for roofing and glass cullet from a CRT were used. The CRT glass was obtained from the front screen part of the CRT. The part of the picture tube from which the glass cullet is obtained determines its chemical composition. The chemical composition of the glass in the screen part is different than in the neck of the picture tube, because in addition to the basic elements that make up the glass structure, it also contains Ba and Sr, while in the neck of the picture tube and in the funnel, there is lead oxide, which is not present in the screen part.

Each of the materials used in the vitrification process was subjected to chemical and phase composition tests. The chemical composition tests were performed employing the WD-XRF spectroscopic method by means of an Axios Max WD-XRF X-ray fluorescence spectrometer (Malvern Panalytical Ltd., Malvern, UK) with wavelength dispersion using a 4 kW Rh lamp. The chemical composition of ACW and CRT cullet is shown in Table 1. X-ray structural studies were performed utilizing a Philips X’Pert Pro X-ray diffractometer (Koninklijke Philips N.V., Amsterdam, The Netherlands) with a copper anode lamp. A measurement step of 0.008° and a pulse counting time of 50 s were used. The phases were identified based on data from the ICDD PDF-4 database.

**Figure 1:**
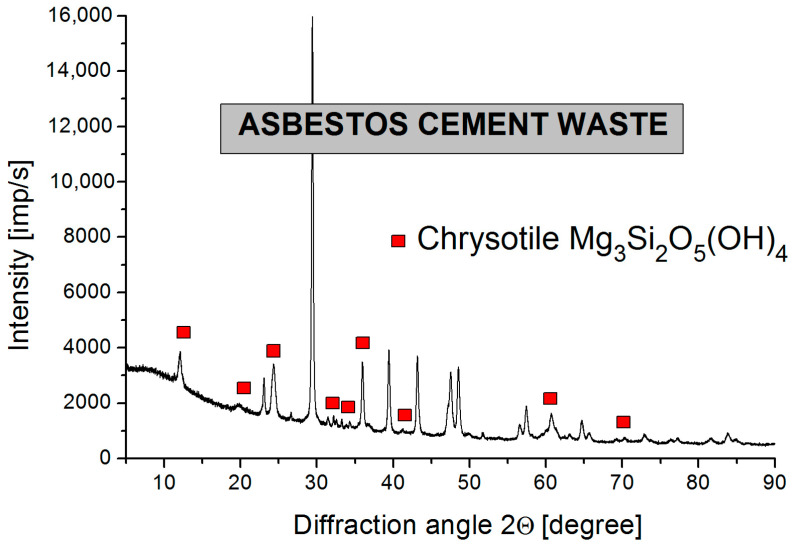
Diffractogram of ACW.

The glass from the CRT contained, apart from the main component, i.e., silicon, significant amounts of barium and strontium, but no lead was found in it. This composition proves that the CRT screen is made of silicate glass with an increased content of barium and strontium. X-ray examinations of the CRT glass revealed a complete lack of reflections from crystalline phases, which is characteristic of amorphous materials. In the case of the asbestos-cement waste, the X-ray structural tests showed the presence of chrysotile—Mg_3_Si_2_O_5_(OH)_4_. The Eternit also contained calcite (CaCO_3_), portlandite (Ca(OH)_2_) and larnite (Ca_2_SiO_4_). Detailed XRD studies of ACW are presented in the article [11]. A diffractogram of ACW is shown in Figure 1.

**Figure 2:**
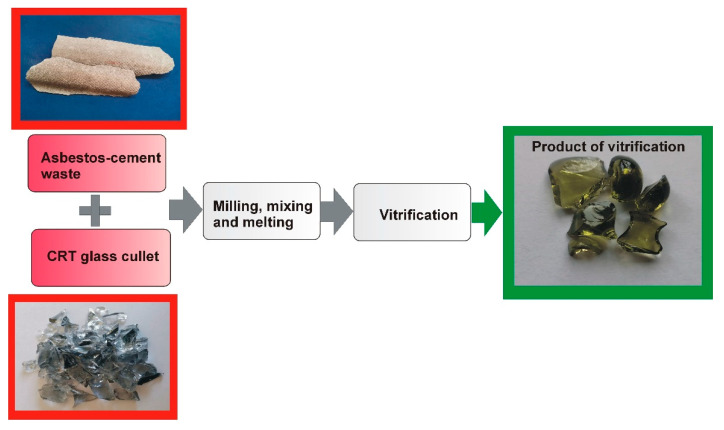
Scheme of producing vitrified material from asbestos-cement waste and CRT glass cullet.

The glass cullet and asbestos-cement waste were initially crushed and then ground for 5 min in a FRITSCH Pulverisette 6 planetary mill (FRITSCH GmbH, Idar-Oberstein, Germany). The rotational speed of the grinding bowl was 300 rpm. Grinding definitely facilitated thorough mixing of both the ingredients in the assumed weight proportions, which in this case was 50:50. The raw material set prepared in this way was placed in a porcelain crucible and melted in an electric furnace at a temperature of 1400 °C for 90 min. The molten material was poured onto a cold steel plate to induce vitrification. The produced vitrified material was then annealed at a temperature of 700 °C, and then cooled with the furnace to ambient temperature. The scheme of producing vitrified material from asbestos-cement waste and CRT glass cullet is shown in Figure 2.

The vitrified material was subjected to X-ray structural and WD-XRF spectroscopic tests and hardness measurements. The chemical composition of vitrified material is shown in Table 2. The chemical composition of the vitrified material is a compilation of the chemical compositions of the components involved in its formation and evidences the migration of these components into the emerging structure of the vitrified material.

**Figure 3:**
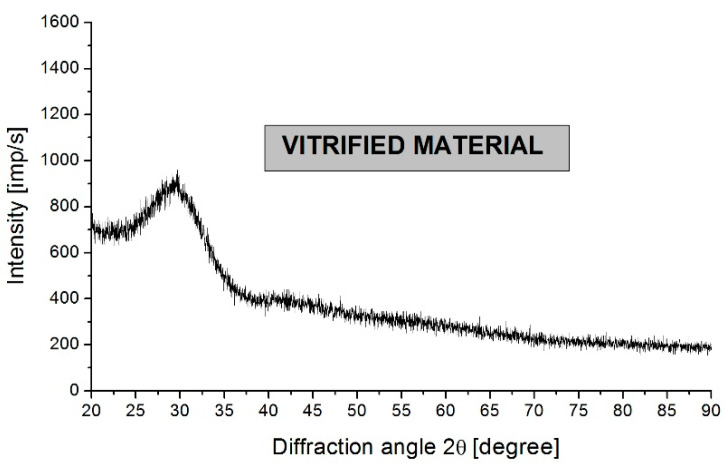
Diffractogram of vitrified material.

The X-ray analysis showed a complete lack of reflections from crystalline phases, and only the presence of a characteristic amorphous halo, i.e., a broad peak of low intensity in the angular range of 25°–35°. This nature of the diffractogram proves the amorphous structure of the vitrified material. A diffractogram of vitrified material is shown in Figure 3.

Hardness measurements were carried out using a Shimatzu HMV-G20 microhardness tester (Shimatzu Corp., Kyoto, Japan) equipped with a Vickers penetrator. A load of 1.961 N was applied and the loading time was 10 s. Both the CRT glass and the vitrified material were subjected to comparative tests. The hardness of the CRT glass was 596 HV0.2 and that of the vitrification product was 637 HV0.2. As can be seen, the hardness of the vitrified material was almost 7% higher than the hardness of the CRT glass used in its production. Both materials had an amorphous structure, but differed in their chemical composition, which in this case is responsible for the differences found in hardness. The higher hardness of the vitrified material compared to CRT glass is mainly due to the increased content of oxides such as Al_2_O_3_, ZrO_2_ and MgO and a lower share of modifying oxides, i.e., Na_2_O and K_2_O [16].

## 3. Methodology of Composite Production

The composite was produced by the FSP method developed by Mishra and Mahoney [17], derived from the FSW method (friction stir welding) patented by Thomas et al. from the Welding Institute [18]. The FSP method uses a special cylindrical tool, most often equipped with a pin and a shoulder. During FSP and FSW, the tool is set in rotation, and after the material has plasticized, it moves at a set speed in the assumed direction, in accordance with the adopted machining strategy [19,20]. Plasticization of the matrix material is possible owing to the heat generated as a result of intense friction of the tool against the sample surface and strong plastic deformation of the material.

The mixing efficiency and intensity of material flow, and therefore the final effects of FSP, are determined by the dimensions of the pin and the shoulder, their shape and surface profile, but also by the rotational speed of the tool and its traverse speed. In the case of composite production, the task of the tool is not only to plasticize the matrix, but also to mix the individual components that make up the composite. In the FSP hole variant, the material constituting the reinforcing phase is placed in separate chambers hollowed out in the surface of the material comprising the matrix of the future composite. These holes act as reservoirs from which the reinforcing phase is then introduced into the plasticized composite matrix.

In this work, an original hole solution with a shifted pin working zone was used. The essence of this solution is that in the first phase of processing, the tool does not move along the line defined by the holes hollowed out in the sample surface as is the case with the standard solution, but is spaced from it by a certain constant value ΔL (in this work, ΔL was 2.5 mm). In the second phase of processing, the tool moves along the primary line of the holes and this stage is carried out in order to obtain a more even distribution of the reinforcing phase in the matrix. Details of this solution and the benefits resulting from its use are discussed in the paper [21].

**Figure 4:**
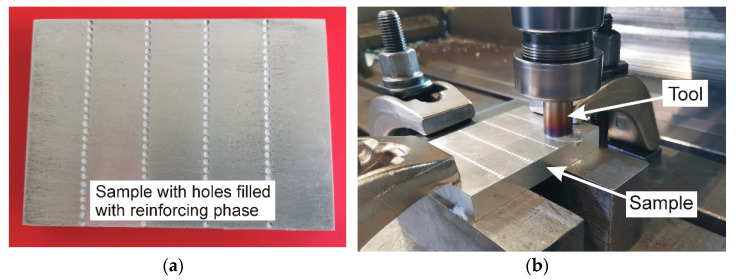
(**a**) Sample after filling with reinforcing phase; (**b**) friction stir processing of AA7075 aluminum alloy.

In this work, a tool with a flat shoulder surface and a conical pin with a threaded side surface was utilized. The pin length was 5 mm, and the diameter of the shoulder was 18 mm. The tool was made of hot work tool steel X37CrMoV5-1. The tool traverse speed was set at 30 mm/min and the rotational speed at 400 rpm. The reinforcing material was placed in holes with a diameter of 2 mm and a depth of 4.6 mm (Figure 4a). The composite manufacturing process is shown in Figure 4b. The produced composite was subjected to microstructural tests, hardness and wear resistance measurements, and based on the obtained results, the suitability of the product received from the vitrification of ACW and CRT waste as a reinforcing phase in the composite was assessed.

## 4. Results and Discussion

### 4.1. Microstructural Studies

**Figure 5:**
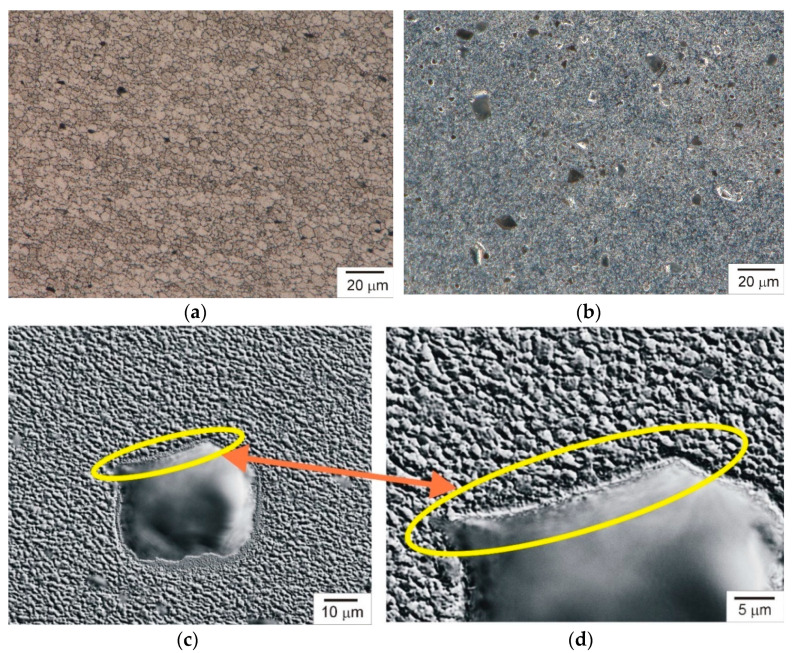
(**a**) Microstructure of stirred zone; (**b**) composite reinforced with vitrified material; (**c**,**d**) inter-phase boundary in composite reinforced with vitrified material. Light microscopy. Optical shadow effect mode (**c**,**d**).

Light microscopy examinations were performed by means of a Keyence VHX-7000 light microscope (Keyence Ltd., Osaka, Japan). As a result of FSP, a composite surface layer of approximately 5 mm was obtained in the AA7075 alloy surface layer, i.e., a thickness approximately corresponding to the length of the pin of the tool used in treatment. The reinforcing phase in the form of vitrified particles was located mainly in the stirred zone, i.e., the central, dominant part of the friction-modified zone in terms of dimensions. During FSP or FSW, the material in the stirred zone is subjected to the strongest plastic deformation and the highest temperature, resulting in the presence of very fine equiaxed grains in this zone, which indicates the formation of the material microstructure under dynamic recrystallization conditions. Strong grain refinement, apart from the presence of the reinforcing phase, is the main component of the microstructural changes found in the stirred zone (Figure 5a,b). The average grain size in the stirred zone was approximately 2 µm, almost 150 times smaller than the average grain size in the initial alloy, which was 290 µm.

Microscopic examination of the friction-stir-processed layer also showed that after FSP, the rolling structure present in the starting material was eliminated, the distribution of intermetallic phase precipitates became more uniform and there was no banding. Moreover, the processing resulted in partial dissolution of these phases in the matrix, and those that were not dissolved were redistributed while retaining their original shape and size.

One of the most important issues in the production of composite materials is the even distribution of the reinforcing phase particles in the matrix. The performed tests showed that the particles of the reinforcing phase in the produced composites were separated from each other by the matrix and were mostly evenly distributed in the matrix, as can be seen in Figure 5b. When producing composites, it is also important that the particles of the reinforcing phase are well connected to the matrix. If the particles of the reinforcing phase are isolated from the matrix, such particles are easily chipped, e.g., when the composite is operated under abrasive wear conditions. The tests carried out revealed that the particles of the vitrified material were well bonded in the matrix alloy, as evidenced by the microstructures presented in Figure 5c,d. The presence of particle agglomerates was not detected either.

### 4.2. Hardness Measurements

One of the main goals of producing composite surface layers is to increase the hardness and improve the tribological properties of the layer while maintaining the core properties. The hardness was measured on sample sections at a load of 980.7 mN. The time of applying pressure was 10 s.

**Figure 6:**
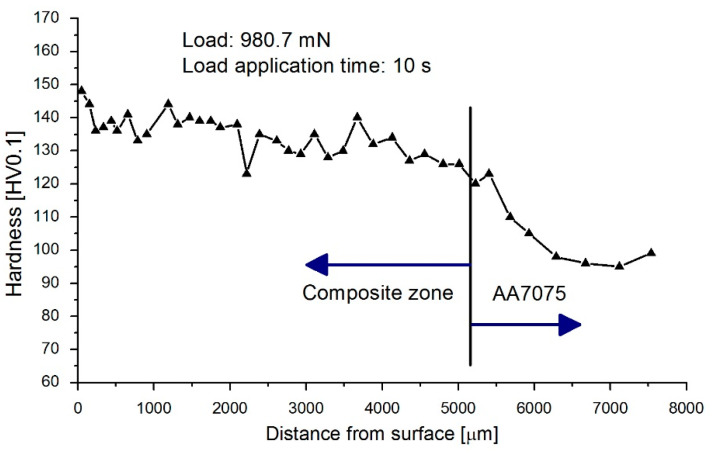
Hardness measurement results.

The hardness tests revealed clearly higher hardness of the composite reinforced with the vitrification product compared to the AA7075 alloy. The hardness of the aluminum alloy was 97 HV0.1, while the average hardness of the composite reinforced with the vitrified material was 135 HV0.1, which is approximately 39% higher. Examples of graphical dependencies of hardness as a function of distance from the surface are shown in Figure 6.

When assessing the hardness of the composite and the reasons for the increase in this hardness, two main factors should be taken into account, namely, the degree of grain refinement, and above all, the presence of hard particles of the reinforcing phase. The influence of the first of these factors is described by the so-called Hall–Petch law. This law shows the relationship between the grain size and material hardness [22,23]. The above relationship shows that the hardness is proportional to *d*^−1/2^, where *d* is the average grain diameter; thus, the smaller the grain, the greater the hardness of the material. This is because grain refinement is accompanied by an increment in the total number of grain boundaries, which in turn constitute a barrier to dislocations and cause their immobilization. This mechanism is the basis of so-called grain boundary strengthening or Hall–Petch strengthening [24,25].

### 4.3. Wear Resistance Tests

**Figure 7:**
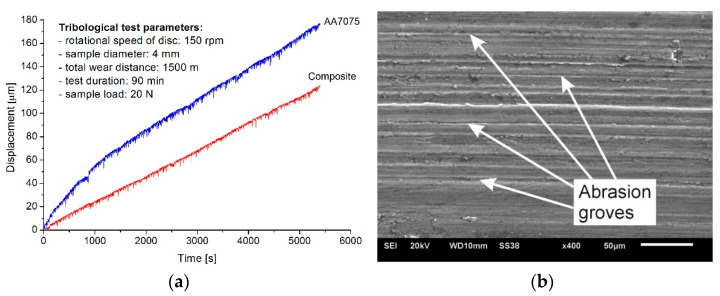
(**a**) Results of tribological tests; (**b**) worn surfaces of composite sample (SEM).

The last stage of the research verifying the suitability of the vitrification product as a reinforcing phase in the composite concerned wear resistance. Tests of the material wear resistance were performed utilizing a pin-on-disc T-01M tribological tester (ITEE, Radom, Poland) under dry friction conditions. The samples had the form of a pin with a diameter of 4 mm, while the counter-sample had the form of a ring with an outer diameter of 42 mm. The counter-sample was made of bearing steel EN 100Cr6 (E52100 according to AISI). The disk rotation speed was 150 rpm. The load applied during the test was constant and amounted to 20 N. The tribological test lasted 106 min, and the total wear distance was 1500 m. During the test, a linear change in the sample length as a function of time was recorded. The results of the tribological tests are shown in Figure 7a.

Tribological tests make it possible to analyze the friction process and assess the resistance of the tested material to friction wear, which is one of the key criteria for predicting the application potential of the tested material. The tribological tests showed that introducing vitrified material particles into the AA7075 alloy matrix results in a 30.4% smaller loss of the tested sample compared to its counterpart without the reinforcing phase.

As in the case of hardness, two main factors are responsible for improving wear resistance, namely, high grain refinement and the presence of hard reinforcing particles in the aluminum alloy matrix. The particles of the reinforcing phase reduce the contact surface of the tested sample with the counter-sample in the friction node, which results in lower wear intensity of its surface.

After the tribological tests, the samples were subjected to microscopic analysis to identify the wear mechanism. These studies were performed using a JEOL JSM-6610LV scanning electron microscope (JEOL Ltd., Tokyo, Japan). Based on the nature of the changes occurring in the geometric structure of the surface, the mechanism of material wear can be determined. As can be seen in Figure 7b, the dominant effects occurring on the surface of the composite samples were mutually parallel scratches resulting from micro-cutting and micro-plowing, which indicates the occurrence of abrasive wear of the material during the test.

## 5. Conclusions

Based on the conducted research, the following statements and conclusions were formulated:

1. The product of the vitrification of asbestos-cement waste and glass cullet from a CRT can be used as a reinforcing phase in the production of composites and constitutes a substitute material for other currently used materials.
2. The introduction of vitrified particles into the AA7075 aluminum alloy matrix, combined with strong grain refinement, raised the hardness of the material by approximately 39%, from 97 HV0.1 to approximately 135 HV0.1.
3. Favorable changes in the microstructure of the material increase the abrasive wear resistance of the vitrified-material-reinforced composite by 30.4% compared to the wear resistance of the AA7075 aluminum alloy.
4. The use of vitrified material as a reinforcing phase allows a high-quality surface composite to be obtained. The use of the vitrified material as a strengthening phase in composites is therefore fully justified and advisable.

## Figures and Tables

**Table 1:** Chemical composition of ACW and CRT cullet.

Sample	Element Content (wt%)											
	CaO	SiO_2_	MgO	Al_2_O_3_	Fe_2_O_3_	BaO	SrO	SO_3_	K_2_O	Na_2_O	ZrO_2_	Rest
ACW	69.92	14.80	4.15	2.47	4.44	-	-	2.57	0.27	-	-	1.38 *
CRT glass cullet	0.10	60.49	0.05	3.05	0.06	8.37	7.77	0.13	6.24	9.49	2.57	1.68 **

* (P_2_O_3_, Cl, TiO_2_, Cr_2_O_3_, MnO, NiO, CuO, ZnO, Ga_2_O_3_, GeO_2_). ** (P_2_O_3_, Cl, TiO_2_, Cr_2_O_3_, MnO, NiO, CuO, ZnO, Ga_2_O_3_, Rb_2_O, Y_2_O_3_, Sb_2_O_3_, Cs_2_O, CeO_2_, HfO_2_).

**Table 2:** Chemical composition of vitrified material.

Sample	Element Content (wt%)											
	CaO	SiO_2_	MgO	Al_2_O_3_	Fe_2_O_3_	BaO	SrO	SO_3_	K_2_O	Na_2_O	ZrO_2_	Rest
Vitrified material	34.01	29.52	1.59	3.91	2.31	5.86	11.09	0.19	3.37	1.80	3.88	2.47 *

* (P_2_O_5_, TiO_2_, NiO, CuO, ZnO, Ga_2_O_3_, GeO_2_, Y_2_O_3_, Sb_2_O_3_, CeO_2_).

## Data Availability

The data that support the findings of this study are available from the corresponding author, J. Iwaszko, upon reasonable request.
